# Targeting Multiple Mitochondrial Processes by a Metabolic Modulator Prevents Sarcopenia and Cognitive Decline in SAMP8 Mice

**DOI:** 10.3389/fphar.2020.01171

**Published:** 2020-07-31

**Authors:** Dario Brunetti, Emanuela Bottani, Agnese Segala, Silvia Marchet, Fabio Rossi, Fiorenza Orlando, Marco Malavolta, Michele O. Carruba, Costanza Lamperti, Mauro Provinciali, Enzo Nisoli, Alessandra Valerio

**Affiliations:** ^1^ Department of Medical Biotechnology and Translational Medicine, University of Milan, Milan, Italy; ^2^ Medical Genetics and Neurogenetics Unit, Fondazione IRCCS Istituto Neurologico C. Besta, Milan, Italy; ^3^ Department of Molecular and Translational Medicine, University of Brescia, Brescia, Italy; ^4^ Department of Diagnostics and Public Health, University of Verona, Verona, Italy; ^5^ Advanced Technology Center for Aging Research, Scientific Technological Area, IRCCS INRCA, Ancona, Italy; ^6^ Center for Study and Research on Obesity, University of Milan, Milan, Italy

**Keywords:** essential amino acids, aging, sarcopenia, cognitive impairment, tricarboxylic acid cycle, mitochondrial respiratory chain, mitochondrial biogenesis, proteostasis

## Abstract

The age-dependent declines of skeletal muscle and cognitive functions often coexist in elderly subjects. The underlying pathophysiological mechanisms share common features of mitochondrial dysfunction, which plays a central role in the development of overt sarcopenia and/or dementia. Dietary supplementation with formulations of essential and branched-chain amino acids (EAA-BCAA) is a promising preventive strategy because it can preserve mitochondrial biogenesis and function. The senescence-accelerated mouse prone 8 (SAMP8) is considered an accurate model of age-related muscular and cognitive alterations. Hence, we aimed to investigate the progression of mitochondrial dysfunctions during muscular and cognitive aging of SAMP8 mice and to study the effects of a novel EAA-BCAA-based metabolic modulator on these changes. We evaluated body condition, motor endurance, and working memory of SAMP8 mice at 5, 9, 12, and 15 months of age. Parallel changes in protein levels of mitochondrial respiratory chain subunits, regulators of mitochondrial biogenesis and dynamics, and the antioxidant response, as well as respiratory complex activities, were measured in the quadriceps femoris and the hippocampus. The same variables were assessed in 12-month-old SAMP8 mice that had received dietary supplementation with the novel EAA-BCAA formulation, containing tricarboxylic acid cycle intermediates and co-factors (PD-0E7, 1.5 mg/kg/body weight/day in drinking water) for 3 months. Contrary to untreated mice, which had a significant molecular and phenotypic impairment, PD-0E7-treated mice showed preserved healthy body condition, muscle weight to body weight ratio, motor endurance, and working memory at 12 months of age. The PD-0E7 mixture increased the protein levels and the enzymatic activities of mitochondrial complex I, II, and IV and the expression of proliferator-activated receptor γ coactivator-1α, optic atrophy protein 1, and nuclear factor, erythroid 2 like 2 in muscles and hippocampi. The mitochondrial amyloid-β-degrading pitrilysin metallopeptidase 1 was upregulated, while amyloid precursor protein was reduced in the hippocampi of PD-0E7 treated mice. In conclusion, we show that a dietary supplement tailored to boost mitochondrial respiration preserves skeletal muscle and hippocampal mitochondrial quality control and health. When administered at the early onset of age-related physical and cognitive decline, this novel metabolic inducer counteracts the deleterious effects of precocious aging in both domains.

## Introduction

Aging is a natural multifactorial process characterized by a gradual decrease in physiological functions, resulting in reduced resistance to stress, high vulnerability to diseases, and increased probability of death. Although physical difficulties can occur independently of cognitive decline, physical deterioration often coexists with cognitive impairment in the aged population, with a consequent erosion of older people’s independence (Gómez-Gómez and Zapico, 2019). The frequent association of physical and cognitive decline has recently led to the proposal of the so-called “motoric-cognitive risk” (MCR) syndrome (Verghese et al., 2013), which may affect up to 10% of older persons worldwide (Maggio and Lauretani, 2019). Whether affected subjects are at higher risk of dementia remains a matter of debate.

Sarcopenia, recently recognized as a disease by the World Health Organization (Anker et al., 2016), is an age-associated loss of skeletal muscle mass and function, leading to reduced physical performance (Cruz-Jentoft and Sayer, 2019). It is independently associated with comorbidities, including cognitive impairment (Hsu et al., 2014). Sarcopenia is recognized as a core feature of frailty syndrome (Cruz-Jentoft and Sayer, 2019), a condition of decreased physiological reserve mostly affecting the skeletal muscle and the brain, besides other organs and systems, and leading to increased vulnerability to adverse health outcomes (Clegg et al., 2013). Of note, frailty has been recently found to enhance the risk for clinically relevant dementia in the presence of Alzheimer’s disease (AD)-related brain pathology (Wallace et al., 2019). The prevention of sarcopenia and cognitive decline is a significant area of current research as a result of their increasing prevalence in a globally aging world. Therefore, it would be useful to investigate the shared pathophysiological mechanism(s) of physical and cognitive decline as potential target(s) for preventive or therapeutic interventions.

Mitochondria are mainly responsible for energy generation and hold a central position in cellular homeostasis, driving many aspects of the biological aging process. Several studies pointed toward a central role of mitochondria in aging, especially in post-mitotic, highly energy-demanding tissues like heart, skeletal muscle, and brain (Nilsson et al., 2019). Mitochondrial dysfunction is a crucial pathological mechanism involved in the etiology of sarcopenia. Several mitochondrial abnormalities have been reported in age-related muscular atrophy and weakness, including accumulation of mitochondrial DNA (mtDNA) damage, impaired mitochondrial biogenesis, dynamics, and quality control as well as reduced mitochondrial bioenergetics, and increased oxidative stress (Rygiel et al., 2016). Similarly, age-related decrease in mitochondrial bioenergetics, which is paralleled by increased reactive oxidative species (ROS) in the animal and human brain, has been linked to a deterioration of cognitive functions and the onset of dementia (Grimm et al., 2016; Cenini et al., 2019). Extensive research purports that mitochondrial derangements contribute to AD pathogenesis or possibly even initiate pathologic molecular cascades upstream of amyloid-β (Aβ) deposition in AD (Swerdlow, 2018).

Rejuvenating strategies to counteract the aging process and favor healthy aging (i.e., aging without related illness) have been investigated for many years; over the past decade, numerous studies have identified fundamental mechanisms of aging, along with targeted interventions that temper those mechanisms in laboratory animal models. In this context, targeting mitochondria is considered an attractive strategy to modulate the aging process (Valerio and Nisoli, 2015; Madreiter-Sokolowski et al., 2018). We have previously demonstrated that a specific mixture of essential amino acids (EAAs) enriched in branched-chain amino acids (BCAA-enriched mixture, BCAAem) activated the eNOS/mTORC1/PGC-1α cascade in middle-aged mice, so preserving cardiac and skeletal muscle mitochondrial biogenesis, enhancing physical endurance and increasing average lifespan (D’Antona et al., 2010). Subsequently, we showed that BCAAem was able to protect mice from rosuvastatin-induced myopathy (D’Antona et al., 2016) and to increase oxidative fiber content and ameliorate muscular dystrophy phenotype in mutant *mdx* mice (Banfi et al., 2018). Multiple mechanisms contribute to BCAAem-mediated skeletal muscle protection, including the rescue of *de-novo* protein synthesis and reduction of protein breakdown, and notably, improvement of mitochondrial function and enhancement of ROS defense system (D’Antona et al., 2016; Banfi et al., 2018). Following human pilot studies, dietary supplementation with the BCAAem formula is emerging as a promising treatment option in elderly subjects (Aquilani et al., 2011; Aquilani et al., 2019). Most recently, with a randomized trial on 155 elderly malnourished patients (MATeR study), we demonstrated the ability of BCAAem supplementation to improve general health, muscle mass, and strength, as well as cognitive performance (Buondonno et al., 2019). This study emerged that BCAAem-mediated health benefits were accompanied by an increase of mitochondrial bioenergetics in peripheral blood mononuclear cells of elderly patients (Buondonno et al., 2019).

We investigated whether a novel BCAA-enriched EAA formulation, with a balanced BCAA stoichiometric ratio (leucine:isoleucine:valine 2:1:1) and added Krebs’ cycle precursors and co-factors (referred to as PD-0E7), could boost oxidative metabolism and contrast age-related mitochondrial dysfunction in skeletal muscle and brain. To this aim, we used the senescence accelerated mouse prone 8 (SAMP8) model, which progressively develops sarcopenia (An Yun et al., 2015) and cognitive declines with an Alzheimer–like phenotype (Akiguchi et al., 2017) between 9 and 12 months of age. We analyzed the effects of PD-0E7 on motor and cognitive functions and on mitochondrial parameters in skeletal muscles and hippocampi. Overall, our findings demonstrate that i) pharmacological modulation of mitochondrial metabolism with a nutraceutical intervention is a feasible approach *in vivo*, and ii) the correction of mitochondrial dysfunction counteracts the onset of age-related cognitive and muscular decline, opening new perspectives for the achievement of healthy aging.

## Materials and Methods

### Animals and Dietary Supplementation

All experiments were performed in accordance with the European Community Council Directive 2010/63/EU. According to current Italian law (D. Lgs. n. 26/2014), the protocol was approved by the General Direction of Animal Health and Veterinary Drugs of the Italian Ministry of Health with the authorization n. 131/2018-PR. Male SAMP8 mice (SAMP8/AKR/J, ENVIGO) were housed under specific pathogen-free (SPF) conditions with room temperature set at 22 ± 2°C and a 12-h light-dark cycle, with *ad* *libitum* access to food (Rodents standard diet cat. 4RF25, Mucedola, Settimo Milanese, Milan, Italy) and water. The experimental design included four groups (n=6 mice/group) of untreated mice aged 5, 9, 12, and 15 months. Another group of 9-month-old mice (n = 12) was randomly divided in two experimental groups, unsupplemented, and PD-0E7-supplemented (n=6/each), the latter receiving the PD-0E7 for 3 months (1.5 mg/g body weight/day in drinking water). The composition of the PD-0E7 supplement (Professional Dietetics S.p.A, Milan, Italy) is detailed in **Table S1**. The PD-0E7 dose was selected based on our previous experience with the BCAAem formula (D’Antona et al., 2010) and of the safety and efficiency of a peculiar EAA-BCAA formula containing Krebs’ cycle precursors and co-factors (i.e., citric, succinic, and malic acids with the same percent composition of PD-0E7) (Tedesco et al., 2020). A decade of knowledge shows that BCAA-based mixtures are safe in both rodents and humans and, in particular, do not harm the liver nor the kidney (Valerio et al., 2011; Tedesco et al., 2018). The amount of PD-0E7 to be dissolved in water was calculated by recording the average body weight and the average daily water consumption of each experimental group for 2 weeks before starting the treatment, and regularly adjusted based on the same parameters. Body weight was measured once a week, and water intake was checked twice a week. Fresh PD-0E7 was replaced three times per week. Mice were culled by cervical dislocation. Tissues were immediately harvested and differentially stored according to the subsequent analysis. For histochemical analysis, tissues were frozen in liquid-nitrogen pre-cooled isopentane and stored at −80°C; for biochemical assays, blue native gel electrophoresis (BNGE), and Western blot analysis, samples were snap-frozen in liquid nitrogen and stored at −80°C.

### Chemicals

All chemicals and reagents were purchased from Sigma Aldrich (Milan, Italy), unless otherwise stated.

### Health Status and Behavioral Analysis

Mice were monitored weekly for general health or behavioral changes and onset of postural abnormalities. Body condition score (BCS) was assessed according to an established method (Ullman-Culleré and Foltz, 1999). Kyphosis, a characteristic dorsal curvature of the spine (frequently due to backbone muscle weakness in elderly people and progeroid mice models) (Harkema et al., 2016), was assessed by visual inspection. The presence of hind limb clasping reflex (a common manifestation of neurological or neurodegenerative disease in mice) (Lalonde and Strazielle, 2011) was assessed suspending the mice by the tail for 20 s and observing if they clasped their hind limbs together; normal mice splay their limbs apart. Motor skills were evaluated measuring endurance as previously described (Viscomi et al., 2011) using a treadmill apparatus (Ugo Basile, Italy) with a gradually accelerating protocol (the speed was initially set at 3.8 m/min and increased by 3 m/min every 2 min). The test was terminated by exhaustion, which was defined by 10 falls/min into the motivational grid. The cognitive status of SAMP8 mice was evaluated by measuring spontaneous alternation in the Y-maze, a test assessing the immediate spatial working memory, which strongly relies on the hippocampus and prefrontal cortex functions (Hölter et al., 2015). The Y-maze apparatus consists of three arms at equal angles (30 cm length × 5 cm width × 12 cm height). In this test, each mouse was placed in the center of the maze facing toward one of the arms and then allowed to explore freely for 6 min. An arm entry was counted when the hind paws of the mouse were completely into the arm. The series of arm entries were visually recorded, and the percentage of alternation was calculated. A spontaneous alternation was defined as successive entries into the three arms. The percentage of alternation (Y-maze score) was calculated by the following equation: % alternations = [(number of spontaneous alternations)/(total arm entries − 2)] × 100 (Hölter et al., 2015).

### Histochemistry and Biochemical Assays

Histochemical analysis of cytochrome c oxidase (COX, complex IV) in mouse gastrocnemius muscle was carried out on 8-µm cryostat sections as previously described (Sciacco and Bonilla, 1996). For biochemical analyses, mouse quadriceps samples were homogenized in 10 mM phosphate buffer (pH 7.4). Enzyme activities of individual respiratory chain complexes were measured spectrophotometrically as previously described in detail (Bugiani et al., 2004).

### Isolation of Mitochondria and Blue Native Gel Electrophoresis

Mitochondria from skeletal muscles were isolated by differential centrifugation (Fernández-Vizarra et al., 2002) and resuspended in 25 mM sucrose, 75 mM sorbitol, 100 mM KCl, 0.05 mM EDTA, 5 mM MgCl_2_, 10 mM Tris–HCl (pH 7.4), and 10 mM H_3_PO_4_, pH 7.4 (Civiletto et al., 2015). For BNGE analysis, 100 μg of mitochondria were resuspended in NativePAGE buffer (Invitrogen), protease inhibitors, and 4% digitonin and incubated for 1 h on ice before centrifuging at 20,000 g at 4°C. Coomassie G250 at 5% final concentration was added to the supernatants. Proteins (30 μg aliquots) were separated by pre-cast NativePAGE 3–12% Bis-Tris gels (Thermo Fisher Scientific) according to the manufacturer’s protocol and electroblotted on polyvinylidene fluoride (PVDF) membranes for immunodetection (Civiletto et al., 2015).

### Western Blot Analysis

Mouse *quadriceps femoris* and hippocampi were homogenized in ten volumes of radioimmunoprecipitation assay (RIPA) buffer [150 mM NaCl, 5 mM EDTA, 50 mM Tris, 1% NP-40, 0.5% sodium deoxycholate, and 0.1% sodium dodecyl sulfate (SDS)] in the presence of protease and phosphatase inhibitors, with the use of automatic homogenizer (Omni international, Kennesaw, GA). Samples were incubated on ice for 30 min and centrifuged at 14,000 x g for 20 min at 4°C. Protein concentration was determined with the BCA protein assay Kit (Pierce Biotechnology, Rockford, IL). Aliquots (20 μg each) were run through a 4–12% SDS–polyacrylamide gel electrophoresis (PAGE), electroblotted onto a PVDF or nitrocellulose membrane and probed with different primary antibodies: total oxidative phosphorylation (OXPHOS) rodent antibody cocktail (Abcam, ab110413, dilution 1:1,000); OPA1 (Immunological Sciences, 1:1,000); PITRM1 (ATLAS, 1:1,000); APP (Abcam, 1:1,000); GAPDH (Immunological Sciences, 1:2,000), PGC-1α (Novus Biological, 1:1,000), p70 S6 kinase and phospho-p70 S6 kinase (Cell Signaling Technology 1:1,000), SIRT1 (Cell Signaling Technology 1:1,000), Nrf2 (BioSource, 1:500), IDE (Abcam 1:1,000), chemiluminescence-based immunostaining (ECL Western Blotting Detection Kit, Amersham) was performed. Images were acquired with the use of ChemiDoc Imaging System apparatus (Bio-Rad Laboratories, Milan, Italy) and analyzed with Image Lab™ software, version 6.0.1 for Windows (Bio-Rad Laboratories, Milan, Italy).

### Statistical Analysis

One-way ANOVA with Tukey’s post-test for multiple comparison or Student’s t-test were used to compare the means among four age groups or between two groups, respectively. Fisher Exact test was performed to evaluate significance of frequency records among four age groups. The data were analyzed using GraphPad Prism version 5.00 for Windows (GraphPad Software, San Diego, CA). A linear model fitted using generalized estimating equation (GEE) accounting for the within-subject correlation was used to analyze longitudinal experiments, either with Gaussian error distribution for quantitative data or with binomial distribution for binary events. Models were fitted using R (version 3.6.3). All tests were two-sided and assumed a 5% significance. See text or figure legends for details.

## Results

### SAMP8 Mice Show Age-Related Physical and Cognitive Decline

**Figure 1:**
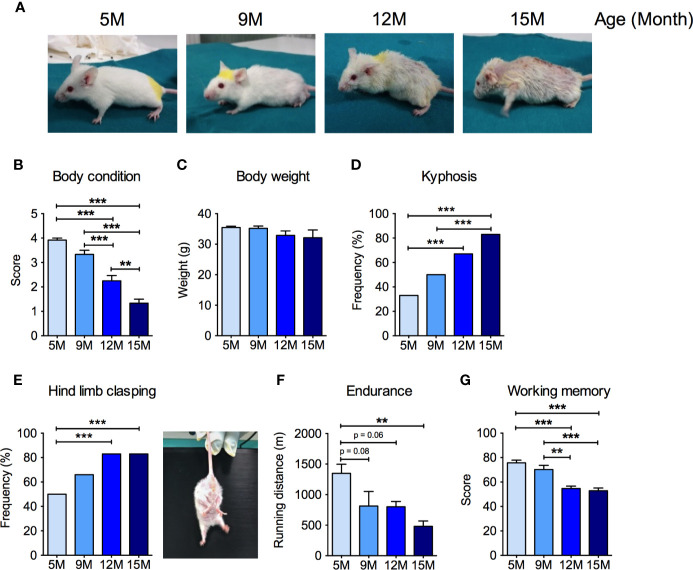
Neuromuscular and cognitive phenotypical characterization of SAMP8 mice at different ages. **(A)** Representative pictures of SAMP8 mice at 5, 9, 12, and 15 months of age show a progressive appearance of signs of aging, which were quantified by means of body condition score **(B)**, body weight **(C)**, frequency of kyphosis **(D)**, and frequency of hind limb clasping reflex **(E)**. SAMP8 mice also showed a progressive reduction in exercise endurance measured by treadmill exhaustion test **(F)**, which was paralleled by a decline of working memory quantified by Y-maze test **(G)**. Statistical analyses were performed with one-way ANOVA and Tukey’s multiple comparison test **(B**, **C**, **F**, **G)** or Fisher test **(D**, **E)**. Error bars represent SEM; n=6 mice/group; **p < 0.01, ***p < 0.001.

We first performed a comprehensive characterization of age-related changes in the general health status, as well as in motor and cognitive abilities in male SAMP8 mice at 5, 9, 12, and 15 months of age. SAMP8 mice showed an age-dependent decrease of the BCS (Ullman-Culleré and Foltz, 1999) (**Figures 1A, B**). In particular, at 5 months of age, SAMP8 mice appeared healthy and well-conditioned, without prominence of vertebrae and dorsal pelvis; conversely, in the 9- and 12-month-old groups, mice progressively appeared under-conditioned, with evident segmentation of vertebral column and palpable dorsal pelvic bones. At 15 months of age, SAMP8 mice looked emaciated, with an extremely prominent skeletal structure (**Figure 1A**). We observed a non-significant decrease in the body weight along with mice age (**Figure 1C**) without changes in food and water intake (not shown). SAMP8 mice showed an ever-increasing prevalence of kyphosis that became statistically significant in the 12-month-old group (**Figure 1D**), with further intensification at 15 months, suggesting a progressive impairment of back muscle mass and strength. Exercise endurance, as assessed by the capacity to run until exhaustion during a treadmill test, gradually and significantly diminished with age (**Figure 1E**). SAMP8 mice also revealed an age-related increase in the frequency of the hind limb clasping reflex (an index of disease progression in various mouse models of brain lesions and neurodegeneration, including AD) (Lalonde and Strazielle, 2011) (**Figure 1F**). Further, as quantified by the Y maze score (**Figure 1G**), we found a substantial age-dependent reduction of the spatial working memory (one of the early memory deficits in human AD and AD mouse models) (Webster et al., 2014). Taken together, these data indicate that SAMP8 mice developed an age-related impairment of skeletal muscle function, along with neurological and cognitive deterioration.

### SAMP8 Mice Show Age-Related Mitochondrial Dysfunction in Skeletal Muscle

**Figure 2:**
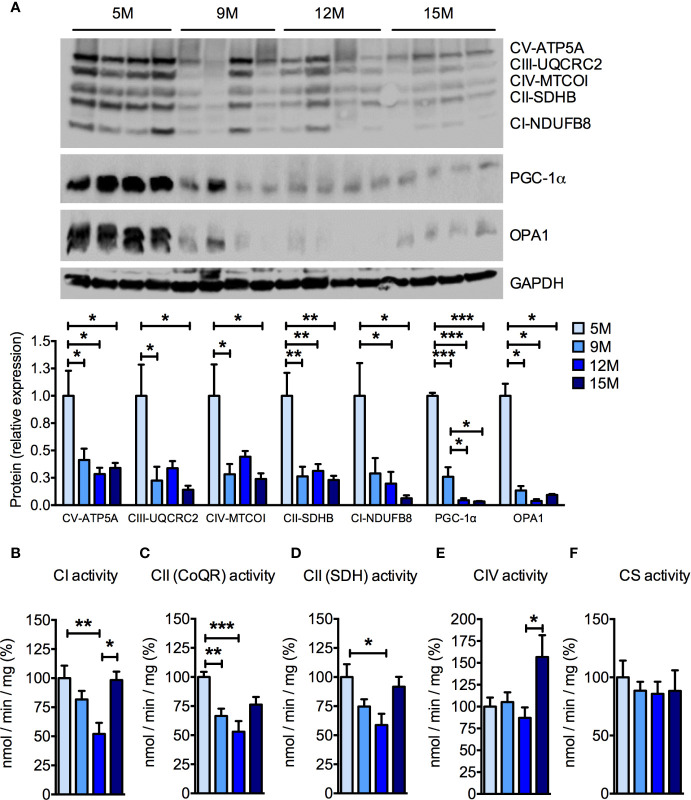
Age-dependent mitochondrial dysfunction in the skeletal muscle of SAMP8 mice. **(A)** Western blot analysis of the mitochondrial respiratory chain (MRC) subunits ATP5A (complex V), UQCRC2 (complex III), MTCOI (complex IV), SDHB (complex II), and NDUFB8 (complex I) at different ages. The quantification of each signal was normalized to the amount of GAPDH and represented below the images. **(B**–**F)** Enzymatic activities of MRC complex I (CI), succinate-coenzyme Q reductase (CoQR) and succinate dehydrogenase (SDH) activities of complex II (CII), complex IV (CIV), and citrate synthase activity were measured at different ages. All color codes as in **(A)**. Statistical analysis was performed with one-way ANOVA and Tukey’s multiple comparison test. Error bars represent SEM; n=4 mice/group **(A)** and n=6 mice/group **(B**–**F)**; *p < 0.05, **p < 0.01, ***p < 0.001.

Because muscular mitochondrial function declines with age (Rygiel et al., 2016), we analyzed mitochondrial function in the skeletal muscles of the four groups of SAMP8 mice. Significant, age-related reductions of the protein levels of several subunits of the oxidative phosphorylation pathway were detected by Western blot analysis (**Figure 2A**) of *quadriceps femoris*. In particular, the amount of the nuclear-encoded subunits of complex I (NDUFB8, NADH:ubiquinone oxidoreductase subunit B8), complex II (SDHB, succinate dehydrogenase complex iron sulfur subunit B), complex III (UQCRC2, ubiquinol-cytochrome C reductase core protein 2), and complex V (ATP5A, ATP synthase F1 subunit alpha), as well as the mitochondrially-encoded subunit of complex IV (MTCOI, mitochondrial cytochrome c oxidase subunit 1) were reduced by more than 50% from 9 months of age onwards (**Figure 2A**). As previously observed in aged C57BL/6J mice (Houtkooper et al., 2011), the master transcriptional regulator of mitochondrial biogenesis peroxisome proliferator-activated receptor γ coactivator-1α (PGC-1α) was reduced in skeletal muscle of aged SAMP8 mice (**Figure 2A**). The mitochondrial dynamin-like GTPase optic atrophy protein 1 (OPA1), which is required for mitochondrial fusion, cristae remodeling, and mitochondrial respiration (Del Dotto et al., 2018) also decreased along with age in SAMP8 *quadriceps femoris* (**Figure 2A**). Accordingly, the enzymatic activities of individual mitochondrial respiratory chain (MRC) complexes were also reduced when measured by spectrophotometric assays (**Figures 2B–E**). In particular, the specific activity of complex I (**Figure 2B**) and both the oxidation of succinate (**Figure 2C**) and the reduction of ubiquinone (coenzyme Q) to ubiquinol (**Figure 2D**), carried out by complex II, were decreased in the 9- and 12-month-old mice compared to the 5-month-old mice. Interestingly, complex I enzymatic activity seemingly recovered in the 15-month-old mice (**Figure 2B**), whereas complex IV activity was not affected until the 15 months of age, when it was significantly increased (**Figure 2E**). No differences were found in the specific activity of citrate synthase (CS) (**Figure 2F**), suggesting that the observed differences were not due to a significant loss of mitochondrial mass. These data confirm that mitochondrial deterioration is a key hallmark of aging in the SAMP8 mouse model.

### SAMP8 Mice Show Age-Related Mitochondrial Dysfunction and Impaired APP Metabolism in Hippocampus

**Figure 3:**
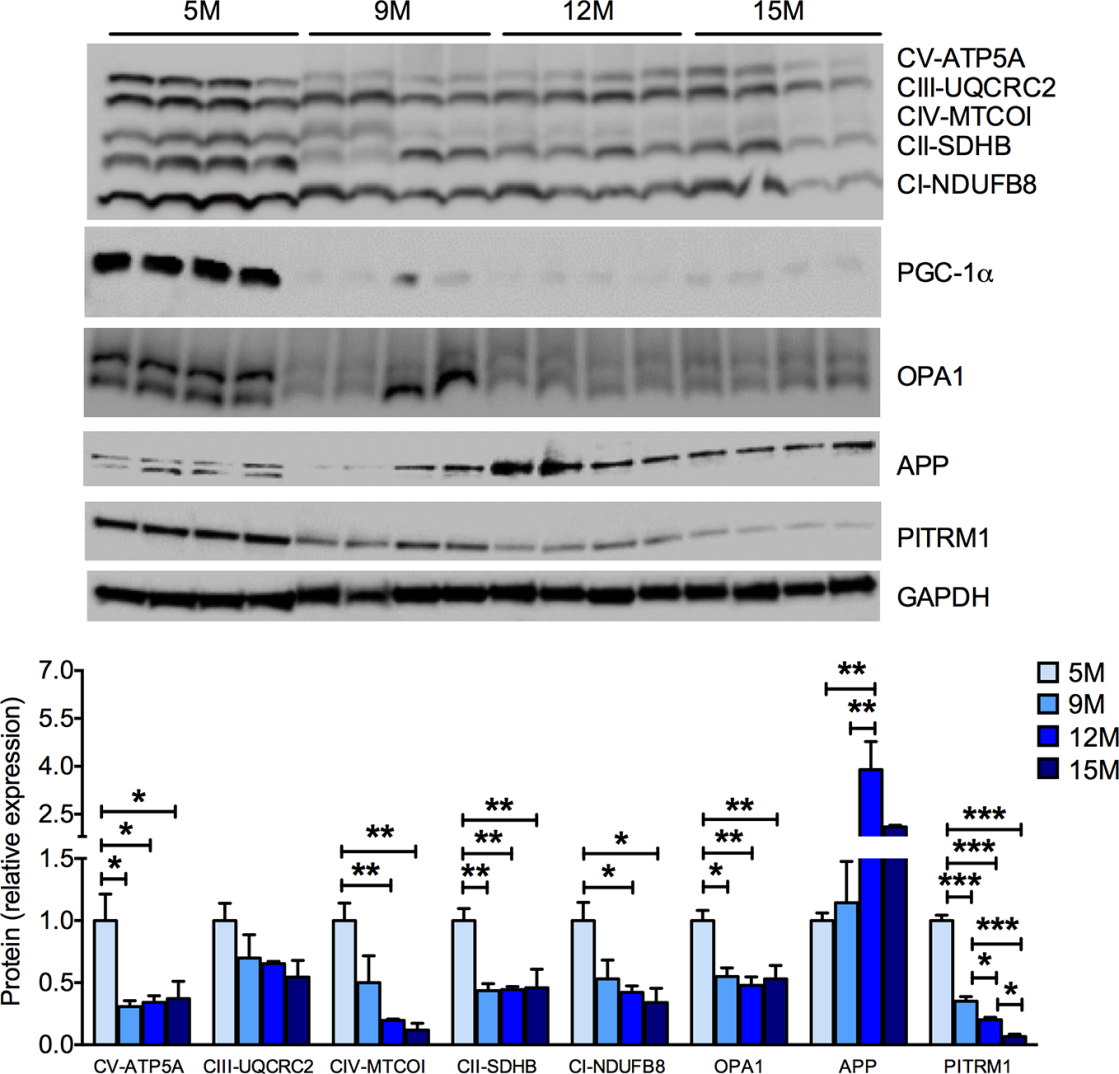
Progressive mitochondrial dysfunction and impaired proteostasis in the hippocampus of SAMP8 mice. Western blot analysis of hippocampal homogenates shows an age-dependent decrease of the mitochondrial respiratory chain (MRC) subunits ATP5A (complex V or ATP synthase), UQCRC2 (complex III), MT-COI (complex IV), SDHB (complex II), and NDUFB8 (complex I). The mitochondrial-shaping protein OPA1 was also reduced with aging. In parallel, SAMP8 mice hippocampi showed a significant increase of amyloid precursor protein (APP) and a decrease of the pitrilysin metallopeptidase 1 (PITRM1) protein levels. The quantification of each signal per group were normalized to the amount of GAPDH and represented below the Western blot images. Statistical analysis was performed with one-way ANOVA and Tukey’s multiple comparison test; error bars represent SEM; n=4 mice/group; *p < 0.05, **p < 0.01, ***p < 0.001.

As the age-related cognitive impairment observed in SAMP8 mice (**Figure 1G**) may rely on hippocampal dysfunction (Pallas et al., 2008), we investigated the mitochondrial function in the hippocampus of SAMP8 mice. As detected in the skeletal muscles, a significant, age-related decrease in the amount of MRC subunits was found in hippocampal homogenates (**Figure 3**). The protein levels of PGC-1α and of OPA1, which are reduced in AD brain (Qin et al., 2009; Wang et al., 2009), were also reduced along with age in the SAMP8 hippocampus (**Figure 3**). Altered amyloid precursor protein (APP) metabolism, a crucial facet of brain aging, has been previously described in the hippocampi of SAMP8 mice (Pallas et al., 2008). As mitochondria are the sites in which Aβ peptides are partially processed (Mossmann et al., 2014; Brunetti et al., 2016), we investigated whether APP level changes would match the mitochondrial dysfunction in the aged hippocampi. We found an age-dependent increase of APP levels in SAMP8 hippocampi (**Figure 3**). Interestingly, this phenomenon was paralleled by a decrease in the levels of the pitrilysin metallopeptidase 1 (PITRM1) (**Figure 3**). PITRM1 is responsible for the degradation of the mitochondrial fraction of Aβ, and the impairment of its activity results in Aβ accumulation and the backlogging of its precursor, further leading to amyloidotic neurodegeneration (Brunetti et al., 2016; Langer et al., 2018). Together, these data support a mitochondrial dysfunction in the onset of the age-associated cognitive decline of SAMP8 mice.

### PD-0E7 Supplementation Prevents Age-Related Physical and Cognitive Decline

**Figure 4:**
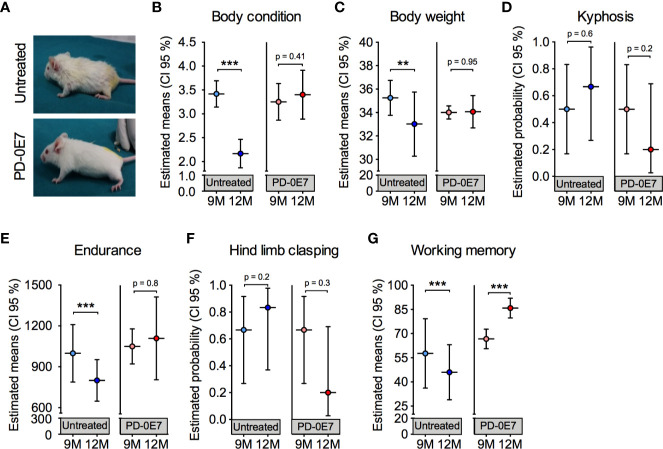
Effect of PD-0E7 supplementation on muscular and cognitive function in aged SAMP8 mice. **(A)** Representative pictures of 12-month-old untreated and PD-0E7-treated mice. The efficacy of the PD-0E7 was investigated in a longitudinal experiment comparing the behavioral scores of single mice at baseline (9M, 9 months of age) and after 3 months of supplementation (12M, 12 months of age). Control, untreated mice were also individually scored at baseline (9M) and after 3 months (12M). Treatment effects across time for body condition score **(B)**, body weight **(C)**, endurance capacity **(E)**, and working memory **(G)** means were analyzed with generalized estimating equation (GEE) with Gaussian error distribution. Frequency of kyphosis **(D)** and hind limb clasping **(F)** data were analyzed with GEE with binomial distribution. Error bars represent 95% confidence interval; n=6 mice/untreated group; 5 mice/PD-0E7-treated group, due to one death after baseline assessment. **p < 0.01; ***p < 0.001.

In a diverse set of experiments, we tested whether dietary supplementation with the PD-0E7 formula was able to counteract the deterioration of general health status and of motor and cognitive abilities in aged mice. Nine-month-old male SAMP8 mice (n = 12) underwent clinical phenotyping at baseline, as described above. Mice were then randomly divided into two experimental groups (n=6/each), either unsupplemented or supplemented with PD-0E7 for 3 months. At the end of the experimental protocol, a second clinical phenotyping was performed. PD-E07 dietary supplementation did not affect food consumption and slightly but not significantly increased water consumption in SAMP8 mice (**Table S2**). Twelve-month-old, PD-0E7-treated mice appeared healthier and better conditioned than the age-matched untreated mice, which showed prominent kyphosis and hyposthenia of fore- and hindlimb muscles (**Figure 4A**); moreover, PD-0E7-treated mice had thick, glossy fur which was instead lost and replaced by thinning fur in untreated animals (**Figure 4A**). Contrary to untreated mice, PD-0E7-treated mice retained their BCS (time x treatment interaction, p < 0.001; GEE) (**Figure 4B**) and their body weight (time x treatment interaction, p = 0.047, GEE) (**Figure 4C**) for the duration of the treatment. The frequency of kyphosis (**Figure 4D**) and hind limb clasping (**Figure 4F**) showed non-significant trends toward a reduction in PD-0E7-treated mice (time x treatment interaction, p = 0.2 and p = 0.128, respectively; GEE with binomial distribution). While the endurance performance on the treadmill test was significantly reduced in untreated mice, it remained unchanged after the 3 months PD-0E7 treatment (time x treatment interaction, p=0.25; GEE) (**Figure 4E**). Finally, contrary to untreated mice displaying significantly reduced working memory scores at 12 months of age, we recorded a higher working memory score upon PD-0E7 supplementation (time x treatment interaction, p < 0.001; GEE) (**Figure 4G**). On the whole, these results demonstrate the efficacy of the dietary supplementation with the PD-0E7 mixture in counteracting age-associated muscular and cognitive decline.

### PD-0E7 Treatment Preserves Mitochondrial Biogenesis and Oxidative Phosphorylation in the Skeletal Muscles of SAMP8 Mice

**Figure 5:**
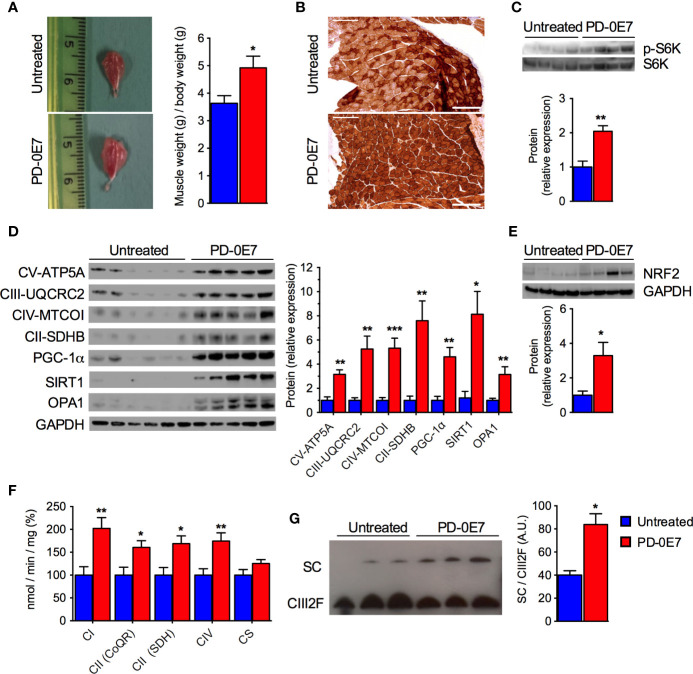
Effect of PD-0E7 on mitochondrial function in skeletal muscle of aged SAMP8 mice. **(A)** Representative pictures of *gastrocnemius* muscles of untreated and PD-0E7-treated mice; quantification of muscle weight/body weight is shown on the right (blue bars, untreated mice, red bars, PD-0E7-treated mice). **(B)** Representative image of histochemical COX-IV staining in the *gastrocnemius* muscle of untreated and PD-0E7-treated SAMP8 mice. **(C)** Western blot analysis of phosphorylated p70 S6 kinase 1 (p-S6K) and total S6K in *quadriceps femoris*; quantification of pS6K/S6K ratio is shown below the image. **(D)** Western blot analysis of PGC-1α, SIRT1, OPA1, and of the respiratory chain subunits ATP5A, UQCRC2, MTCOI, and SDHB in *quadriceps femoris*; relative quantification of proteins to GAPDH levels is shown on the right. **(E)** Western blot analysis of NRF2-ARE in *quadriceps femoris*, with quantification relative to GAPDH shown below the image. **(F)** Biochemical analysis of the enzymatic activities of the mitochondrial respiratory chain (MRC) complex I (CI), complex II (CII), and IV (CIV) in *quadriceps femoris* PD-0E7 treated mice compared to untreated controls. **(G)** Blue native gel electrophoresis on isolated, digitonin-solubilized mitochondria from *quadriceps femoris* of untreated, and PD-0E7-treated mice showed a stabilization of the complex III into supercomplexes (SC); immunoblot was performed with UQCRFS1 antibody; quantification is shown on the right. All color codes as in **(A)**. Statistical analysis was performed with Student’s t test. Error bars represent SEM; n=6 mice/group; *p < 0.05, **p < 0.01, ***p < 0.001.

*Post-mortem* examination of the skeletal muscles showed that PD-0E7-treated SAMP8 mice had significantly higher *gastrocnemius* muscle mass than the untreated group (**Figure 5A**), supporting its efficacy in reducing sarcopenia. Western blot analysis on total homogenates of *quadriceps femoris* showed that the amount of phosphorylated p70 S6 kinase 1 (S6K1) was significantly augmented in the PD-0E7-treated group compared to unsupplemented controls (**Figure 5C**), demonstrating the activation of the mTORC1/S6K1 pathway (Ma and Blenis, 2009), as we reported previously with the *in vivo* administration of the BCAAem formula (D’Antona et al., 2010). We also previously found evidence that BCAAem-activated mTOR signaling enhances PGC-1α-mediated mitochondrial biogenesis (D’Antona et al., 2010). Accordingly, the protein amount of PGC-1α was significantly increased in *quadriceps femoris* from PD-0E7-treated animals compared to untreated controls (**Figure 5D**), together with the amount of the sirtuin family deacetylase SIRT1, which is known to promote PGC-1α activation and expression in the skeletal muscle (Amat et al., 2009) (**Figure 5D**), both suggesting that the mitochondrial biogenesis pathway was activated upon PD-0E7 supplementation. The amounts of the nuclear factor, erythroid 2 like 2 (NFE2L2 or Nrf2-ARE), which regulates the expression of antioxidant genes and is involved in a mitochondrial biogenic regulatory loop involving PGC-1α (Gureev et al., 2019) was also increased in *quadriceps femoris* from PD-0E7-treated animals (**Figure 5E**). The protein content of several respiratory chain subunits, *i.e.*, ATP5A, UQCRC2, MTCOI, and SDHB, were significantly higher than those detected in untreated mice (**Figure 5D**) along with the enzymatic activities of MRC complexes I, II, and IV spectrophotometrically measured in total homogenates (**Figure 5F**). The latter was also confirmed by increased COX reaction at the *gastrocnemius* muscle histochemical staining (**Figure 5B**). Notably, skeletal muscles of the PD-0E7-treated group also showed increased levels of OPA1 (**Figure 5D**), which, independently from its role in mitochondrial fusion, also acts as mitochondrial cristae shaping remodeler and a stabilizer of MRC supercomplexes (SCs), thus impacting on respiratory efficiency (Cogliati et al., 2013). We therefore analyzed SC assembly status in the skeletal muscle of PD-0E7-treated *versus* untreated mice and observed a shift of the free form of the complex III toward functional SCs (**Figure 5G**). Altogether, these data strongly support a role of PD-0E7 in promoting mitochondrial biogenesis, oxidative phosphorylation, and antioxidant response through the mTORC1/S6K1/PGC-1α pathway in the skeletal muscle of SAMP8 mice.

### PD-0E7 Treatment Preserves Mitochondrial Biogenesis and Proteostasis in the Brain of SAMP8 Mice

**Figure 6:**
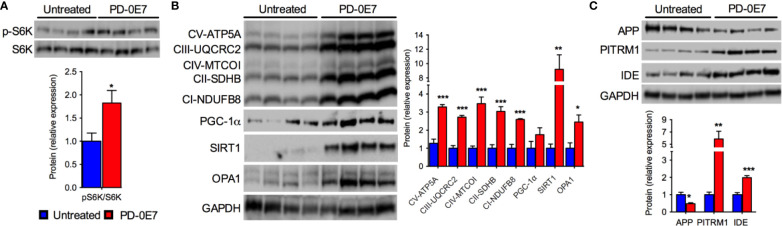
Effects of PD-0E7 on mitochondrial function in the hippocampus of aged SAMP8 mice. **(A)** Western blot analysis of phosphorylated p70 S6 kinase 1 (p-S6K) and total S6K in hippocampal homogenates; quantification of pS6K/S6K ratio is shown below the image (blue bars, untreated mice, red bars, PD-0E7-treated mice). **(B)** Western blot analysis of PGC-1α, SIRT1, OPA1, and the mitochondrial respiratory chain (MRC) subunits ATP5A, UQCRC2, MTCOI, SDHB, NDUFB8 in mouse hippocampi; relative quantification of protein to GAPDH levels is shown on the right. **(C)** Western blot analysis of amyloid precursor protein (APP), pitrilysin metallopeptidase 1 (PITRM1), and insulin-degrading enzyme (IDE) protein levels and quantification relative to GAPDH levels. All color codes as in **(A)**. Statistical analysis was performed with unpaired Student’s t test. Error bars represent SEM, n=4 mice/group, *p < 0.05, **p < 0.01, ***p < 0.001.

We further analyzed the hippocampi of PD-0E7-treated SAMP8 mice and untreated controls by immunoblot and, similarly to what we observed in skeletal muscle, we observed the activation of the mTORC1 pathway as demonstrated by the significant increase of the phosphorylated form of the S6K1 protein (**Figure 6A**). The transcriptional coactivator PGC-1α and the deacetylase SIRT1 were significantly upregulated in the hippocampus from PD-0E7-treated mice (**Figure 6B**), with a consistent increase of the levels of several MRC subunits (ATP5A, UQCRC2, MTCOI, SDHB, NDUFB8). The OPA1 protein was also upregulated in the hippocampus of PD-0E7-supplemented SAMP8 mice (**Figure 6B**). Interestingly, treated animals also showed increased levels of the mitochondrial Aβ scavenger PITRM1 and of the insulin-degrading enzyme (IDE), a protease involved in the degradation of Aβ peptides (Kurochkin and Goto, 1994) that can be induced by SIRT1-mediated mechanisms (Corpas et al., 2017) (**Figure 6C**) which was paralleled by a decrease in the amount of APP (**Figure 6C**). Collectively, these data suggest that dietary supplementation with PD-0E7 could increase mitochondrial biogenesis and regulate amyloid proteostasis through the activation of the mTORC1/S6K1/PGC-1α pathway in SAMP8 mice.

## Discussion

In the present study, we provide evidence of the beneficial effect of a novel nutritional intervention, acting as a metabolic inducer, in rescuing age-related motor and cognitive function in senescent mice. The reciprocal links between muscle and brain dysfunctions in aging have recently led to the proposal of the “motoric-cognitive risk” syndrome, affecting 7–10% of non-demented older individuals (Verghese et al., 2013; Maggio and Lauretani, 2019) and strongly predicting the risk of developing various type of dementia, including AD, and physical frailty (Lauretani et al., 2017). Therefore, concurrently targeting the interlinked domains of the pathological “brain-muscle loop” could be crucial for preserving homeostasis and preventing the leading causes of disability in the elderly. There are currently no adequate pharmacological or nutritional interventions for sarcopenia and cognitive decline. We used the SAMP8 mouse strain—which is prone to accelerated aging with impairments affecting both muscle and brain—to investigate the shared pathophysiological mechanism(s) of physical and cognitive decline, with a particular focus on mitochondrial derangements, and to explore the effectiveness of a novel metabolic modulator in preventing these geriatric conditions.

It has been reported that SAMP8 mice spontaneously develop a pre-sarcopenic status at 8 months of age, that results in overt sarcopenia at 10–12 months of age (Sousa-Victor et al., 2014; An Yun et al., 2015), as well as progressive neurodegenerative changes between 6 and 12 months of age, reminiscent of the pathological shift from aging to dementia of sporadic AD (Pallas et al., 2008; Akiguchi et al., 2017). To identify the proper timing of intervention at the early onset of motor and cognitive impairment, we first performed an in-depth phenotypical characterization of the SAMP8 mice, by recording established scores of their general health status, as well as of their motor and cognitive skills, at 5, 9, 12, and 15 months of age. In line with previous studies (Guo et al., 2015; Griñán-Ferré et al., 2018) we observed an age-dependent physical, neuromuscular, and cognitive decline in this mouse strain. In particular, the early signs of inadequate general health conditions were detectable at 9 months of age, with a trend in the reduction of BCS, as well as in the increased frequency of kyphosis (a sign accompanying age-associated loss of muscle mass in mice) (Harkema et al., 2016) and hind limb clasping (a sign of neurological dysfunction also observed in AD animal models) (Lalonde and Strazielle, 2011). These changes were significant at 12 months of age and further worsened with aging. A marked reduction of exercise tolerance was observed as early as at 9 months of age, with a subsequent further decline, while working memory was significantly impaired from the age of 12 months onwards.

By *post-mortem* examination, such deteriorations evolved in parallel with a progressive impairment of the mitochondrial function in critical tissues. The age-dependent reduction of PGC-1α and of MRC subunits was mirrored by the decrease of the activity of complex I, complex II, and complex IV in skeletal muscles. Interestingly, we found that, despite the similar amount of MRC subunits, muscle mitochondria of the 15-month-old group had higher activities of the respiratory chain complexes I and IV than the 12-month-old ones. Of notice, SAMP8 mice are characterized by a shortened life span, with median and maximum life span expectancy of approximately 10 and 16 months, respectively, the latter reached by 20% of the SAMP8 mouse population (Porquet et al., 2013), though possibly influenced by housing and feeding conditions. Therefore, the 15-month-old group represents the long-lived portion of the SAMP8 colony, sharing some similarities with long-lived individuals. In particular, mitochondria from skin biopsies of long-lived humans (i.e., centenarians) are defective but maintain a higher bioenergetic efficiency compared with old individuals, possibly due to increased autophagic processes (Sgarbi et al., 2014). These phenomena could possibly explain the preserved MRC activities in the 15-month-old group. We also identified an age-dependent down-regulation of the mitochondria-shaping protein OPA1 in the *quadriceps femoris* of SAMP8 mice, as reported in muscles of sarcopenic rodents (Ibebunjo et al., 2013) and in muscle biopsies of old subjects, in whom OPA1 reduction and defects of mitochondrial dynamics correlate with muscle mass loss and muscle force drop (Joseph et al., 2012; Tezze et al., 2017).

The brain is a high-energy-demanding tissue, in which age-related mitochondrial dysfunctions are detrimental (Boveris and Navarro, 2008). Of note, a recent large systematic analysis showed altered expression of genes implicated in mitochondrial function in blood and brain areas of patients with mild cognitive impairment (a condition that is often, but not always, a transitional phase from cognitive changes of normal aging to those typically found in dementia) and of AD patients (Li et al., 2018). The so-called “mitochondrial cascade hypothesis” proposes that mitochondrial dysfunction is the primary event in AD pathology. Numerous experiments proved that the accumulation of Aβ in mitochondria begins before the incidence of extracellular deposition (a view that integrates the amyloid cascade hypothesis), and this leads to mitochondrial dysfunction with increased oxidative stress, impaired mitochondrial dynamics and drop in ATP production, with following synaptic dysfunction, apoptosis, and neurodegeneration (Swerdlow, 2018). Other studies sustain that mitochondrial dysfunction initiates upstream of Aβ deposition, suggesting a primary mitochondrial cascade hypothesis (Swerdlow, 2018). Bioenergetic failure, therefore, lies at the core of Alzheimer’s disease pathophysiology.

Although apparently lacking neurofibrillary tangles (one of the pathological hallmarks of AD), SAMP8 mouse brain shows many neuropathological signatures of AD, including APP overproduction, abnormal Aβ accumulation and impaired Aβ clearance, hyperphosphorylation of tau protein, increased oxidative stress, and gliosis, especially in the hippocampus (Pallas et al., 2008; Morley et al., 2012; Akiguchi et al., 2017). These neuropathological changes increase with age and correlate with the early and progressive decline of learning and memory abilities in SAMP8 mice (Akiguchi et al., 2017). Thus, this strain is considered a good preclinical model for studying the earliest neurodegenerative changes triggered by age‐related events and associated with sporadic AD (Akiguchi et al., 2017). In agreement with the various learning and memory deficits reported in SAMP8 mice (Morley et al., 2012), we observed an age-dependent impairment in immediate spatial working memory, which relies on the hippocampus and its connections with the prefrontal cortex (Hölter et al., 2015). We further demonstrated a progressive, significant reduction of the levels of various MRC subunits in the hippocampus, which occurred in parallel to the decrease of PGC-1α and OPA1 levels, as well as to APP accumulation and PITRM1 reduction. PITRM1 (also known as presequence peptidase, PreP) is a mitochondrial matrix enzyme that digests the N-terminal peptides of proteins imported across the inner mitochondrial membrane, after they have been cleaved off from precursor proteins by the mitochondrial matrix peptidase (MMP) (Alikhani et al., 2011). These peptides are amphiphilic species, with a polar, positively charged, arginine-rich side, opposite to an apolar side. If not cleared from the mitochondrial matrix, they may act as detergent-like, toxic agents, forming pores in the membranes dissipating the mitochondrial membrane potential. It was demonstrated that PITRM1 also act as scavenger digesting the mitochondrial fraction of Aβ. Of note, impaired PITRM1 activity has been detected in the brain from transgenic AD mice and in the temporal lobes of AD patients compared with non-AD age-matched controls (Alikhani et al., 2011). Further investigation has confirmed that the accumulation of Aβ exerts a feedback inhibition on MPP and PITRM1 activity, leading to impaired mitochondrial presequence processing and accumulation of precursor proteins, supporting its central role in the age-dependent vicious cycle of mitochondrial dysfunction in AD (Mossmann et al., 2014). We previously demonstrated that a 50% reduction of PITRM1 amount in the hemizygous *PITRM1^+/−^* mouse causes slowly progressive, multisystem neurological impairment, with age-dependent accumulation in the brain of both Aβ42 (the key amyloidogenic Aβ species in AD) and APP (Brunetti et al., 2016). It has been recently also observed that cerebral organoids generated from PITRM1^−/−^ human induced pluripotent stem cells display increased APP levels and Aβ42/Aβ40 ratio, as well as pathological features of AD, including protein aggregation, tau hyperphosphorylation, and neuronal death (Pérez et al., 2020). Our present data showed that the reduction of PITRM1 is paralleled by increased APP and decreased MRC subunit levels in the hippocampus of SAMP8 mice, possibly contributing to mitochondrial dysfunction and to the onset of cognitive symptoms.

A body of research efforts is currently devoted to boosting mitochondrial bioenergetics as novel therapeutic approaches to treat sarcopenia (Waltz et al., 2018), cognitive decline, and AD (Lejri et al., 2019; Perez Ortiz and Swerdlow, 2019). Given that these conditions will continue to rise worldwide alongside with the population aging, a focus on prevention is urgently needed. Studies in preclinical models are necessary to investigate the effects of compounds that could sustain age-related skeletal muscle and brain dysfunctions. We took advantage of our long-standing experience with the BCAAem formula, which preserves skeletal muscle health by targeting mitochondrial function *via* the mTORC1/PGC-1α pathway (D’Antona et al., 2010; D’Antona et al., 2016; Banfi et al., 2018), and has recently proven to be effective in improving muscular and cognitive performance in elderly subjects (Buondonno et al., 2019). We recently demonstrated that adding tricarboxylic acid cycle intermediates and co-factors to an EAA-BCAA mixture increases oxidative metabolism much more than the previous formula and potentiates its protective effects against mitochondrial dysfunction (Tedesco et al., 2020). Here we tested the novel EAA formulation PD-0E7, with an optimized stoichiometric ratio of BCAAs, and added Krebs’ cycle precursors and co-factors, in SAMP8 senescent mice.

We found that supplementation with PD-0E7 in the temporal window between 9- and 12-month of age beneficially affected the whole body and counteracted the age-related signs of neuromuscular and cognitive decline of SAMP8 mice. These effects were accompanied by increased activation of the mTORC1 pathway (as demonstrated by increased S6K1 phosphorylation) both in skeletal muscle and in hippocampus of PD-0E7 treated mice. Of note, mTORC1 activation plays a role in exercise-mediated health benefits on physical and cognitive performance (Watson and Baar, 2014). In particular, increased mTORC1 signaling is responsible for changes in skeletal muscle growth that occur in response to exercise in rodents and humans (Goodman, 2014; Goodman, 2019). In turn, exercise—which in normal mice extends health span (i.e. healthy disease-free life span)—remains the only currently available intervention to mitigate and even reverse the age-related decline in muscle mass and function of sarcopenia. Although reduction of mTORC1 signaling is generally accepted as a fundamental mechanism implied in the pro-longevity effects of multiple strategies, including calorie restriction, intermittent fasting, and drug treatment (e.g., rapamycin), studies with rapamycin and related drugs, or genetic mTORC1 inhibition showed deleterious effects on muscle mass and functions (see Adegoke et al., 2019 and reference herein for review) (Adegoke et al., 2019). Notably, in older people mTORC1 becomes less responsive to contraction-induced activation compared with young adults (Fry et al., 2011). Additionally, exercise upregulates PGC-1α, hence counteracting its age-related decline (Gill et al., 2018). Activation of mTORC1 favors PGC-1α coactivation of its own promoter (Cunningham et al., 2007) and positively correlates with cellular oxidative capacity (Schieke et al., 2006). Accordingly, we found that chronic treatment with PD-0E7 increased PGC-1α protein levels and upregulated several MRC subunits, also enhancing their activity in mouse skeletal muscle and hippocampus. Raised SIRT1 levels in both tissues of PD-0E7-treated mice could further amplify the mitochondrial responses by deacetylating and activating PGC-1α (Dominy et al., 2010). Finally, skeletal muscle of PD-0E7-treated mice displayed augmented levels of Nrf2, a transcription factor that activates antioxidant genes upon binding to antioxidant-responsive elements (ARE) motifs in their promoters (Ma, 2013). Increased mitochondrial respiration can augment ROS production if appropriated mitochondrial quality control lacks. Although optimal levels of ROS act as essential biological signals regulating various physiological processes (Ristow and Schmeisser, 2014), excess ROS levels can damage cellular components contributing to age-related pathology. Notably, both PGC-1α and Nrf2 are involved in mitochondrial biogenesis and in the endogenous antioxidant response, and a positive regulatory loop has been described between the two factors, overall contributing to maintain mitochondrial mass and redox homeostasis (Ryoo and Kwak, 2018). Hence, simultaneous activation of the PGC-1α and Nrf2 pathways is being considered one of the most promising strategies in gerontoprotection (Gureev et al., 2019).

PD-0E7-supplemented mice showed higher muscle weight when compared to the age-matched untreated controls. The preserved exercise endurance observed in the 12-month-old PD-0E7-supplemented mice might be explained by the increased activity of MRC enzymes (CI, CII, and CIV complexes) and by a substantial shift toward oxidative COX-positive fibers in the gastrocnemius muscle. Interestingly, the protein levels of OPA1 were significantly increased in the muscle of PD-0E7-treated group. In addition to its role in mitochondrial fusion, OPA1 regulates mitochondrial cristae remodeling by promoting the juxtaposition of the cristae membranes (Varanita et al., 2015; Del Dotto et al., 2018). Cristae tightness, in turn, determines the assembly and stability of respiratory chain complexes into SC structures (Cogliati et al., 2013). These functional quaternary structures increase the electron flow channeling during respiration, minimizing electron leaks (Acin-Perez and Enriquez, 2014; Enriquez and Lenaz, 2014), and stabilizing individual respiratory chain complexes such as complex III (Acín-Pérez et al., 2004), thus affecting mitochondrial respiratory efficiency. Indeed, OPA1 overexpression optimizes the ATP production and ameliorates mitochondrial dysfunction *in vivo* (Civiletto et al., 2015). The PD-0E7-mediated increase of OPA1 content in skeletal muscles may explain the improved stabilization of the CIII holocomplex into SCs that we detected by BNGE analysis of the *quadriceps femoris* of PD-0E7-treated SAMP8 mice. A similar link between OPA1 levels, SC organization, and muscle mass was recently described in humans (Tezze et al., 2017). Alterations in SCs have been described in brain and muscle during aging, and interestingly, besides increasing individual MRC subunits, exercise affects the stoichiometry of SC formation in old age (Greggio et al., 2017). In particular, exercise training promoted the redistribution of CIII into SCs in *vastus lateralis* muscle of sedentary older subjects (Greggio et al., 2017). Altogether, these observations suggest that PD-E07 might act as an exercise mimetic in elderly people.

Mice having received PD-0E7 supplementation for 3 months not only did not show the decline in short-term working memory observed in untreated animals, but instead showed a significantly higher memory score when compared to their baseline (at 9 months of age) conditions. Interestingly, SIRT1– whose content is reduced in the cortex of aged SAMP8 mice (Pallàs et al., 2008) and increased by PD-0E7 treatment in the hippocampus of 12-month-old SAMP8 mice compared to untreated mice—is critical for maintaining the physiological acquisition and consolidation of short- and long-term hippocampus-dependent memories (Michán et al., 2010). We also provided substantial evidence that PD-0E7 treatment enhanced the hippocampal mitochondrial quality control system upregulating PITRM1 and IDE levels. Both PITRM1 and IDE belong to the pitrilysin M16 family of proteases, and their activity is lost in AD brain (Alikhani et al., 2011; Leal et al., 2013). The PD-0E7 increased PITRM1 and IDE could account for the observed reduction in hippocampal APP content. Remarkably, PGC-1α also controls Aβ degradation through regulation of the IDE gene expression in the brain (Leal et al., 2013). As such, the PD-0E7-mediated induction of PGC-1α could be a strategic chance not only to boost the oxidative phosphorylation, that restores the ATP levels, and to reduce oxidative stress, but also to favor the mitochondrial proteostasis, which is impaired in aging and in neurodegenerative disease (Leal et al., 2013). Similarly, a transgenic AD mouse model overexpressing PITRM1 in neuronal mitochondria had reduced Aβ levels, improvement of mitochondrial health and synaptic density in the cerebral cortex and hippocampus, as well as preserved spatial learning and memory deficits. These findings support the enhancement of PITRM1 expression as a new therapeutic avenue for the treatment of AD (Fang et al., 2015). Enhancing mitochondrial proteostasis in the brain can represent an important therapeutic strategy to delay Aβ-related toxicity (Sorrentino et al., 2017). Notably, the present findings show that an efficient enhancement of PITRM1-mediated brain proteostasis can be accomplished with a nutraceutical intervention, which deserves to be investigated for its potential role in preventing and/or reducing amyloid pathology.

**Figure 7:**
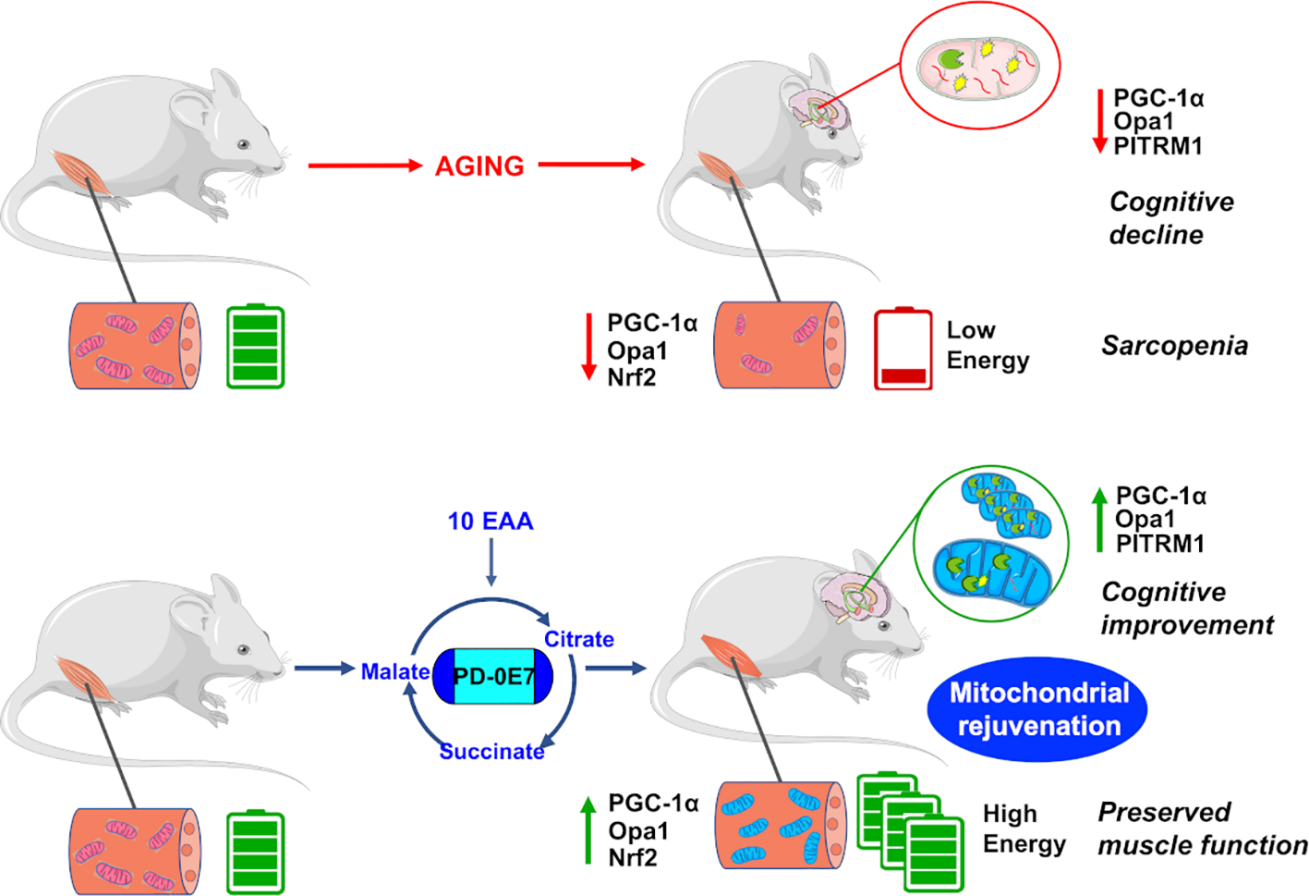
Reduced mitochondrial quality control impairs bioenergetic activity in the skeletal muscles and hippocampus, leading to motor and cognitive dysfunctions in aged mice. Dietary supplementation with the metabolic modulator PD-0E7 preserves mitochondrial efficiency and reverses mice functional decline.

In conclusion, dietary supplementation with the metabolic modulator PD-0E7 rejuvenates mitochondria and efficiently counteracts a plethora of deleterious effects exerted by premature aging in critical tissues (**Figure 7**), thus potentially representing a new efficacious and safe intervention to prevent disability and frailty in subjects at high motoric-cognitive risks.

## Data Availability Statement

The raw data supporting the conclusions of this article will be made available by the authors, without undue reservation.

## Ethics Statement

The animal study was reviewed and approved by the Organismo Preposto al Benessere Animale (OPBA, animal care and health committee) of IRCCS INRCA, Ancona (Italy) and by the Italian Ministry of Health animal committee (authorization n. 131/2018-PR).

## Author Contributions

EN and AV conceived the study. DB and EB made substantial contributions to the design of the study. DB, EB, AS, SM, FR, and FO performed the experiments and analyzed data. MM, CL, MP, EN, and AV analyzed and interpreted data. DB and EB wrote the first draft of the manuscript. DB, EB, AV, and EN wrote the final manuscript. MM, MP, and MC critically revised the manuscript. All authors contributed to the article and approved the submitted version.

## Funding

This work was supported by Fondazione Cariplo (grant #2016-1006 to EN, AV, and MP), by the Italian Ministry of Health (grant RF 2016-02361495 to CL) and partly by Professional Dietetics S.p.A, Milan, Italy. DB and EB were supported by Umberto Veronesi Foundation.

## Conflict of Interest

The authors declare that the research was conducted in the absence of any commercial or financial relationships that could be construed as a potential conflict of interest.

## References

[B1] Acin-Perez R. Enriquez J. A. (2014). The function of the respiratory supercomplexes: The plasticity model. Biochim. Biophys. Acta - Bioenerg. 1837, 444–450. 10.1016/J.BBABIO.2013.12.009 24368156

[B2] Acín-Pérez R. Bayona-Bafaluy M. P. Fernández-Silva P. Moreno-Loshuertos R. Pérez-Martos A. Bruno C. (2004). Respiratory complex III is required to maintain complex I in mammalian mitochondria. Mol. Cell 13, 805–815. 10.1016/s1097-2765(04)00124-8 15053874 PMC3164363

[B3] Adegoke O. A. J. Beatty B. E. Kimball S. R. Wing S. S. (2019). Interactions of the super complexes: When mTORC1 meets the proteasome. Int. J. Biochem. Cell Biol. 117, 105638. 10.1016/j.biocel.2019.105638 31678320 PMC6910232

[B4] Akiguchi I. Pallàs M. Budka H. Akiyama H. Ueno M. Han J. (2017). SAMP8 mice as a neuropathological model of accelerated brain aging and dementia: Toshio Takeda’s legacy and future directions. Neuropathology 37, 293–305. 10.1111/neup.12373 28261874

[B5] Alikhani N. Guo L. Yan S. Du H. Pinho C. M. Chen J. X. (2011). Decreased proteolytic activity of the mitochondrial amyloid-β degrading enzyme, PreP peptidasome, in Alzheimer’s disease brain mitochondria. J. Alzheimers Dis. 27, 75–87. 10.3233/JAD-2011-101716 21750375 PMC3381900

[B6] Amat R. Planavila A. Chen S. L. Iglesias R. Giralt M. Villarroya F. (2009). SIRT1 controls the transcription of the PGC-1α gene in skeletal muscle through PGC-1α auto-regulatory loop and interaction with MyoD. J. Biol. Chem. 284, 21872–21880. 10.1074/jbc.M109.022749 19553684 PMC2755911

[B7] An Yun G. Leung K. S. Siu P. M. F. Qin J. H. Chow S. K. H. Qin L. (2015). Muscle mass, structural and functional investigations of senescence-accelerated mouse P8 (SAMP8). Exp. Anim. 64, 425–433. 10.1538/expanim.15-0025 26193895 PMC4637380

[B8] Anker S. D. Morley J. E. von Haehling S. (2016). Welcome to the ICD-10 code for sarcopenia. J. Cachexia. Sarcopenia Muscle 7, 512–514. 10.1002/jcsm.12147 27891296 PMC5114626

[B9] Aquilani R. Zuccarelli G. C. Dioguardi F. S. Baiardi P. Frustaglia A. Rutili C. (2011). Effects of oral amino acid supplementation on long-term-care-acquired infections in elderly patients. Arch. Gerontol. Geriatr. 52, e123–e128. 10.1016/j.archger.2010.09.005 20934757

[B10] Aquilani R. Zuccarelli Ginetto C. Rutili C. Pisano P. Pasini E. Baldissarro E. (2019). Supplemented amino acids may enhance the walking recovery of elderly subjects after hip fracture surgery. Aging Clin. Exp. Res. 31, 157–160. 10.1007/s40520-018-0941-x 29667153

[B11] Banfi S. D’Antona G. Ruocco C. Meregalli M. Belicchi M. Bella P. (2018). Supplementation with a selective amino acid formula ameliorates muscular dystrophy in mdx mice. Sci. Rep. 8, 14659. 10.1038/s41598-018-32613-w 30279586 PMC6168581

[B12] Boveris A. Navarro A. (2008). Brain mitochondrial dysfunction in aging. IUBMB Life 60, 308–314. 10.1002/iub.46 18421773

[B13] Brunetti D. Torsvik J. Dallabona C. Teixeira P. Sztromwasser P. Fernandez-Vizarra E. (2016). Defective PITRM1 mitochondrial peptidase is associated with A amyloidotic neurodegeneration. EMBO Mol. Med. 8, 176–190. 10.15252/emmm.201505894 26697887 PMC4772954

[B14] Bugiani M. Invernizzi F. Alberio S. Briem E. Lamantea E. Carrara F. (2004). Clinical and molecular findings in children with complex I deficiency. Biochim. Biophys. Acta - Bioenerg. 1659, 136–147. 10.1016/J.BBABIO.2004.09.006 15576045

[B15] Buondonno I. Sassi F. Carignano G. Dutto F. Ferreri C. Pili F. G. (2019). From mitochondria to healthy aging: The role of branched-chain amino acids treatment: MATeR a randomized study. Clin. Nutr. pii: S0261, 33085–33087. 10.1016/j.clnu.2019.10.013 31672329

[B16] Cenini G. Lloret A. Cascella R. (2019). Oxidative Stress in Neurodegenerative Diseases: From a Mitochondrial Point of View. Oxid. Med. Cell. Longev. 2019, 2105607. 10.1155/2019/2105607 31210837 PMC6532273

[B17] Civiletto G. Varanita T. Cerutti R. Gorletta T. Barbaro S. Marchet S. (2015). Opa1 overexpression ameliorates the phenotype of two mitochondrial disease mouse models. Cell Metab. 21, 845–854. 10.1016/j.cmet.2015.04.016 26039449 PMC4457891

[B18] Clegg A. Young J. Iliffe S. Rikkert M. O. Rockwood K. (2013). Frailty in elderly people. Lancet 381, 752–762. 10.1016/S0140-6736(12)62167-9 23395245 PMC4098658

[B19] Cogliati S. Frezza C. Soriano M. E. Varanita T. Quintana-Cabrera R. Corrado M. (2013). Mitochondrial Cristae Shape Determines Respiratory Chain Supercomplexes Assembly and Respiratory Efficiency. Cell 155, 160–171. 10.1016/j.cell.2013.08.032 24055366 PMC3790458

[B20] Corpas R. Revilla S. Ursulet S. Castro-Freire M. Kaliman P. Petegnief V. (2017). SIRT1 Overexpression in Mouse Hippocampus Induces Cognitive Enhancement Through Proteostatic and Neurotrophic Mechanisms. Mol. Neurobiol. 54, 5604–5619. 10.1007/s12035-016-0087-9 27614878

[B21] Cruz-Jentoft A. J. Sayer A. A. (2019). Sarcopenia. Lancet 393, 2636–2646. 10.1016/S0140-6736(19)31138-9 31171417

[B22] Cunningham J. T. Rodgers J. T. Arlow D. H. Vazquez F. Mootha V. K. Puigserver P. (2007). mTOR controls mitochondrial oxidative function through a YY1-PGC-1alpha transcriptional complex. Nature 450, 736–740. 10.1038/nature06322 18046414

[B23] Del Dotto V. Fogazza M. Carelli V. Rugolo M. Zanna C. (2018). Eight human OPA1 isoforms, long and short: What are they for? Biochim. Biophys. Acta - Bioenerg. 1859, 263–269. 10.1016/j.bbabio.2018.01.005 29382469

[B24] Dominy J. E. Lee Y. Gerhart-Hines Z. Puigserver P. (2010). Nutrient-dependent regulation of PGC-1alpha’s acetylation state and metabolic function through the enzymatic activities of Sirt1/GCN5. Biochim. Biophys. Acta 1804, 1676–1683. 10.1016/j.bbapap.2009.11.023 20005308 PMC2886158

[B25] D’Antona G. Ragni M. Cardile A. Tedesco L. Dossena M. Bruttini F. (2010). Branched-chain amino acid supplementation promotes survival and supports cardiac and skeletal muscle mitochondrial biogenesis in middle-aged mice. Cell Metab. 12, 362–372. 10.1016/j.cmet.2010.08.016 20889128

[B26] D’Antona G. Tedesco L. Ruocco C. Corsetti G. Ragni M. Fossati A. (2016). A Peculiar Formula of Essential Amino Acids Prevents Rosuvastatin Myopathy in Mice. Antioxid. Redox Signal. 25, 595–608. 10.1089/ars.2015.6582 27245589 PMC5065032

[B27] Enriquez J. A. Lenaz G. (2014). Coenzyme q and the respiratory chain: coenzyme q pool and mitochondrial supercomplexes. Mol. Syndromol. 5, 119–140. 10.1159/000363364 25126045 PMC4112531

[B28] Fang D. Wang Y. Zhang Z. Du H. Yan S. Sun Q. (2015). Increased neuronal PreP activity reduces Aβ accumulation, attenuates neuroinflammation and improves mitochondrial and synaptic function in Alzheimer disease’s mouse model. Hum. Mol. Genet. 24, 5198–5210. 10.1093/hmg/ddv241 26123488 PMC4550821

[B29] Fernández-Vizarra E. López-Pérez M. J. Enriquez J. A. (2002). Isolation of biogenetically competent mitochondria from mammalian tissues and cultured cells. Methods 26, 292–297. 10.1016/S1046-2023(02)00034-8 12054919

[B30] Fry C. S. Drummond M. J. Glynn E. L. Dickinson J. M. Gundermann D. M. Timmerman K. L. (2011). Aging impairs contraction-induced human skeletal muscle mTORC1 signaling and protein synthesis. Skelet. Muscle. 1, 11. 10.1186/2044-5040-1-11 21798089 PMC3156634

[B31] Gill J. F. Santos G. Schnyder S. Handschin C. (2018). PGC-1α affects aging-related changes in muscle and motor function by modulating specific exercise-mediated changes in old mice. Aging Cell. 17, e12697. 10.1111/acel.12697 PMC5770876 29067788

[B32] Gómez-Gómez M. E. Zapico S. C. (2019). Frailty, Cognitive Decline, Neurodegenerative Diseases and Nutrition Interventions. Int. J. Mol. Sci. 20, 2842. 10.3390/ijms20112842 PMC6600148 31212645

[B33] Goodman C. A. (2014). The role of mTORC1 in regulating protein synthesis and skeletal muscle mass in response to various mechanical stimuli. Rev. Physiol. Biochem. Pharmacol. 166, 43–95. 10.1007/112_2013_17 24442322

[B34] Goodman C. A. (2019). Role of mTORC1 in mechanically induced increases in translation and skeletal muscle mass. J. Appl. Physiol. 127, 581–590. 10.1152/japplphysiol.01011.2018 30676865

[B35] Greggio C. Jha P. Kulkarni S. S. Lagarrigue S. Broskey N. T. Boutant M. (2017). Enhanced Respiratory Chain Supercomplex Formation in Response to Exercise in Human Skeletal Muscle. Cell Metab. 25, 301–311. 10.1016/j.cmet.2016.11.004 27916530

[B36] Grimm A. Friedland K. Eckert A. (2016). Mitochondrial dysfunction: the missing link between aging and sporadic Alzheimer’s disease. Biogerontology 17, 281–296. 10.1007/s10522-015-9618-4 26468143

[B37] Griñán-Ferré C. Corpas R. Puigoriol-Illamola D. Palomera-Ávalos V. Sanfeliu C. Pallàs M. (2018). Understanding Epigenetics in the Neurodegeneration of Alzheimer’s Disease: SAMP8 Mouse Model. J. Alzheimers Dis. 62, 943–963. 10.3233/JAD-170664 29562529 PMC5870033

[B38] Guo A. Y. Leung K. S. Siu P. M. F. Qin J. H. Chow S. K. H. Qin L. (2015). Muscle mass, structural and functional investigations of senescence-accelerated mouse P8 (SAMP8). Exp. Anim. 64, 425–433. 10.1538/expanim.15-0025 26193895 PMC4637380

[B39] Gureev A. P. Shaforostova E. A. Popov V. N. (2019). Regulation of Mitochondrial Biogenesis as a Way for Active Longevity: Interaction Between the Nrf2 and PGC-1α Signaling Pathways. Front. Genet. 10:435:435. 10.3389/fgene.2019.00435 31139208 PMC6527603

[B40] Harkema L. Youssef S. A. de Bruin A. (2016). Pathology of Mouse Models of Accelerated Aging. Vet. Pathol. 53, 366–389. 10.1177/0300985815625169 26864891

[B41] Hölter S. M. Garrett L. Einicke J. Sperling B. Dirscherl P. Zimprich A. (2015). Assessing Cognition in Mice. Curr. Protoc. Mouse Biol. 5, 331–358. 10.1002/9780470942390.mo150068 26629775

[B42] Houtkooper R. H. Argmann C. Houten S. M. Cant́o C. Jeninga E. H. Andreux P. A. (2011). The metabolic footprint of aging in mice. Sci. Rep. 1:134. 10.1038/srep00134 22355651 PMC3216615

[B43] Hsu Y.-H. Liang C.-K. Chou M.-Y. Liao M.-C. Lin Y.-T. Chen L.-K. (2014). Association of cognitive impairment, depressive symptoms and sarcopenia among healthy older men in the veterans retirement community in southern Taiwan: a cross-sectional study. Geriatr. Gerontol. Int. 14 (Suppl 1), 102–108. 10.1111/ggi.12221 24450567

[B44] Ibebunjo C. Chick J. M. Kendall T. Eash J. K. Li C. Zhang Y. (2013). Genomic and proteomic profiling reveals reduced mitochondrial function and disruption of the neuromuscular junction driving rat sarcopenia. Mol. Cell. Biol. 33, 194–212. 10.1128/MCB.01036-12 23109432 PMC3554128

[B45] Joseph A.-M. Adhihetty P. J. Buford T. W. Wohlgemuth S. E. Lees H. A. Nguyen L. M.-D. (2012). The impact of aging on mitochondrial function and biogenesis pathways in skeletal muscle of sedentary high- and low-functioning elderly individuals. Aging Cell 11, 801–809. 10.1111/j.1474-9726.2012.00844.x 22681576 PMC3444680

[B46] Kurochkin I. V. Goto S. (1994). Alzheimer’s beta-amyloid peptide specifically interacts with and is degraded by insulin degrading enzyme. FEBS Lett. 345, 33–37. 10.1016/0014-5793(94)00387-4 8194595

[B47] Lalonde R. Strazielle C. (2011). Brain regions and genes affecting limb-clasping responses. Brain Res. Rev. 67, 252–259. 10.1016/j.brainresrev.2011.02.005 21356243

[B48] Langer Y. Aran A. Gulsuner S. Abu Libdeh B. Renbaum P. Brunetti D. (2018). Mitochondrial PITRM1 peptidase loss-of-function in childhood cerebellar atrophy. J. Med. Genet. 55, 599–606. 10.1136/jmedgenet-2018-105330 29764912

[B49] Lauretani F. Meschi T. Ticinesi A. Maggio M. (2017). “Brain-muscle loop” in the fragility of older persons: from pathophysiology to new organizing models. Aging Clin. Exp. Res. 29, 1305–1311. 10.1007/s40520-017-0729-4 28233284

[B50] Leal M. C. Magnani N. Villordo S. Buslje C. M. Evelson P. Castaño E. M. (2013). Transcriptional regulation of insulin-degrading enzyme modulates mitochondrial amyloid β (Aβ) peptide catabolism and functionality. J. Biol. Chem. 288, 12920–12931. 10.1074/jbc.M112.424820 23525105 PMC3642335

[B51] Lejri I. Agapouda A. Grimm A. Eckert A. (2019). Mitochondria- and Oxidative Stress-Targeting Substances in Cognitive Decline-Related Disorders: From Molecular Mechanisms to Clinical Evidence. Oxid. Med. Cell. Longev. 2019, 1–26. 10.1155/2019/9695412 PMC6535827 31214285

[B52] Li X. Wang H. Long J. Pan G. He T. Anichtchik O. (2018). Systematic Analysis and Biomarker Study for Alzheimer’s Disease. Sci. Rep. 8, 17394. 10.1038/s41598-018-35789-3 30478411 PMC6255913

[B53] Ma X. M. Blenis J. (2009). Molecular mechanisms of mTOR-mediated translational control. Nat. Rev. Mol. Cell Biol. 10, 307–318. 10.1038/nrm2672 19339977

[B54] Ma Q. (2013). Role of Nrf2 in Oxidative Stress and Toxicity. Annu. Rev. Pharmacol. Toxicol. 53, 401–426. 10.1146/annurev-pharmtox-011112-140320 23294312 PMC4680839

[B55] Madreiter-Sokolowski C. T. Sokolowski A. A. Waldeck-Weiermair M. Malli R. Graier W. F. (2018). Targeting mitochondria to counteract age-related cellular dysfunction. Genes (Basel). 9, 165. 10.3390/genes9030165 PMC5867886 29547561

[B56] Maggio M. Lauretani F. (2019). Prevalence, incidence, and clinical impact of cognitive–motoric risk syndrome in Europe, USA, and Japan: facts and numbers update 2019. J. Cachexia. Sarcopenia Muscle 10, 953–955. 10.1002/jcsm.12476 31408280 PMC6818443

[B57] Michán S. Li Y. Chou M. M. H. Parrella E. Ge H. Long J. M. (2010). SIRT1 is essential for normal cognitive function and synaptic plasticity. J. Neurosci. 30, 9695–9707. 10.1523/JNEUROSCI.0027-10.2010 20660252 PMC2921958

[B58] Morley J. E. Armbrecht H. J. Farr S. A. Kumar V. B. (2012). The senescence accelerated mouse (SAMP8) as a model for oxidative stress and Alzheimer’s disease. Biochim. Biophys. Acta - Mol. Basis Dis. 1822, 650–656. 10.1016/J.BBADIS.2011.11.015 22142563

[B59] Mossmann D. Vögtle F.-N. Taskin A. A. Teixeira P. F. Ring J. Burkhart J. M. (2014). Amyloid-β Peptide Induces Mitochondrial Dysfunction by Inhibition of Preprotein Maturation. Cell Metab. 20, 662–669. 10.1016/j.cmet.2014.07.024 25176146

[B60] Nilsson M. II Tarnopolsky M. A. Nilsson M. II Tarnopolsky M. A. (2019). Mitochondria and Aging—The Role of Exercise as a Countermeasure. Biol. (Basel). 8, 40. 10.3390/biology8020040 PMC6627948 31083586

[B61] Pallas M. Camins A. Smith M. A. Perry G. Lee H. Casadesus G. (2008). From Aging to Alzheimer’s Disease: Unveiling “The Switch” with the Senescence-Accelerated Mouse Model (SAMP8). J. Alzheimer’s Dis. 15, 615–624. 10.3233/JAD-2008-15408 19096160

[B62] Pallàs M. Pizarro J. G. Gutierrez-Cuesta J. Crespo-Biel N. Alvira D. Tajes M. (2008). Modulation of SIRT1 expression in different neurodegenerative models and human pathologies. Neuroscience. 154, 1388–1397. 10.1016/j.neuroscience.2008.04.065 18538940

[B63] Pérez M. J. Ivanyuk D. Panagiotakopoulou V. Di Napoli G. Kalb S. Brunetti D. (2020). Loss of function of the mitochondrial peptidase PITRM1 induces proteotoxic stress and Alzheimer’s disease-like pathology in human cerebral organoids. Mol. Psychiatry. 10.1038/s41380-020-0807-4 PMC8758476 32632204

[B64] Perez Ortiz J. M. Swerdlow R. H. (2019). Mitochondrial dysfunction in Alzheimer’s disease: Role in pathogenesis and novel therapeutic opportunities. Br. J. Pharmacol. 176, 3489–3507. 10.1111/bph.14585 30675901 PMC6715612

[B65] Porquet D. Casadesús G. Bayod S. Vicente A. Canudas A. M. Vilaplana J. (2013). Dietary resveratrol prevents Alzheimer’s markers and increases life span in SAMP8. Age (Dordr.) 35, 1851–1865. 10.1007/s11357-012-9489-4 23129026 PMC3776096

[B66] Qin W. Haroutunian V. Katsel P. Cardozo C. P. Ho L. Buxbaum J. D. (2009). PGC-1α expression decreases in the Alzheimer disease brain as a function of dementia. Arch. Neurol. 66, 352–361. 10.1001/archneurol.2008.588 19273754 PMC3052997

[B67] Ristow M. Schmeisser K. (2014). Mitohormesis: Promoting health and lifespan by increased levels of reactive oxygen species (ROS). Dose-Response 12, 288–341. 10.2203/dose-response.13-035.Ristow 24910588 PMC4036400

[B68] Rygiel K. A. Picard M. Turnbull D. M. (2016). The ageing neuromuscular system and sarcopenia: a mitochondrial perspective. J. Physiol. 594, 4499–4512. 10.1113/JP271212 26921061 PMC4983621

[B69] Ryoo I. Kwak M. K. (2018). Regulatory crosstalk between the oxidative stress-related transcription factor Nfe2l2/Nrf2 and mitochondria. Toxicol. Appl. Pharmacol. 359, 24–33. 10.1016/j.taap.2018.09.014 30236989

[B70] Schieke S. M. Phillips D. McCoy J. P. Aponte A. M. Shen R.-F. Balaban R. S. (2006). The mammalian target of rapamycin (mTOR) pathway regulates mitochondrial oxygen consumption and oxidative capacity. J. Biol. Chem. 281, 27643–27652. 10.1074/jbc.M603536200 16847060

[B71] Sciacco M. Bonilla E. (1996). Cytochemistry and immunocytochemistry of mitochondria in tissue sections. Methods Enzymol. 264, 509–521. 10.1016/s0076-6879(96)64045-2 8965723

[B72] Sgarbi G. Matarrese P. Pinti M. Lanzarini C. Ascione B. Gibellini L. (2014). Mitochondria hyperfusion and elevated autophagic activity are key mechanisms for cellular bioenergetic preservation in centenarians. Aging (Albany NY) 6, 296–310. 10.18632/aging.100654 24799450 PMC4032796

[B73] Sorrentino V. Romani M. Mouchiroud L. Beck J. S. Zhang H. D’Amico D. (2017). Enhancing mitochondrial proteostasis reduces amyloid-β proteotoxicity. Nature 552, 187–193. 10.1038/nature25143 29211722 PMC5730497

[B74] Sousa-Victor P. Gutarra S. García-Prat L. Rodriguez-Ubreva J. Ortet L. Ruiz-Bonilla V. (2014). Geriatric muscle stem cells switch reversible quiescence into senescence. Nature 506, 316–321. 10.1038/nature13013 24522534

[B75] Swerdlow R. H. (2018). Mitochondria and Mitochondrial Cascades in Alzheimer’s Disease. J. Alzheimer’s Dis. 62, 1403–1416. 10.3233/JAD-170585 29036828 PMC5869994

[B76] Tedesco L. Corsetti G. Ruocco C. Ragni M. Rossi F. Carruba M. O. (2018). A specific amino acid formula prevents alcoholic liver disease in rodents. Am. J. Physiol. Gastrointest. Liver Physiol. 2018, G566–G582. 10.1152/ajpgi.00231.2017 29368944

[B77] Tedesco L. Rossi F. Ragni M. Ruocco C. Brunetti D. Carruba M. O. (2020). A special amino-acid formula tailored to boosting cell respiration prevents mitochondrial dysfunction and oxidative stress caused by doxorubicin in mouse cardiomyocytes. Nutrients 12, 2020. 10.3390/nu12020282 PMC7071384 31973180

[B78] Tezze C. Romanello V. Desbats M. A. Fadini G. P. Albiero M. Favaro G. (2017). Age-Associated Loss of OPA1 in Muscle Impacts Muscle Mass, Metabolic Homeostasis, Systemic Inflammation, and Epithelial Senescence. Cell Metab. 25, 1374–1389.e6. 10.1016/j.cmet.2017.04.021 28552492 PMC5462533

[B79] Ullman-Culleré M. H. Foltz C. J. (1999). Body Condition Scoring: A Rapid and Accurate Method for Assessing Health Status in Mice. Lab. Anim. Sci. 49, 319–323. 10403450

[B80] Valerio A. Nisoli E. (2015). Nitric oxide, interorganelle communication, and energy flow: a novel route to slow aging. Front. Cell Dev. Biol. 3, 6. 10.3389/fcell.2015.00006 25705617 PMC4319459

[B81] Valerio A. D’Antona G. Nisoli E. (2011). Branched-chain amino acids, mitochondrial biogenesis, and healthspan: an evolutionary perspective. Aging (Albany NY) 3 (5), 464–478. 10.18632/aging.100322 21566257 PMC3156598

[B82] Varanita T. Soriano M. E. Romanello V. Zaglia T. Quintana-Cabrera R. Semenzato M. (2015). The Opa1-dependent mitochondrial cristae remodeling pathway controls atrophic, apoptotic, and ischemic tissue damage. Cell Metab. 21, 834–844. 10.1016/j.cmet.2015.05.007 26039448 PMC4457892

[B83] Verghese J. Wang C. Lipton R. B. Holtzer R. (2013). Motoric cognitive risk syndrome and the risk of dementia. J. Gerontol. - Ser. A Biol. Sci. Med. Sci. 68, 412–418. 10.1093/gerona/gls191 22987797 PMC3593614

[B84] Viscomi C. Bottani E. Civiletto G. Cerutti R. Moggio M. Fagiolari G. (2011). In vivo correction of COX deficiency by activation of the AMPK/PGC-1α axis. Cell Metab. 14, 80–90. 10.1016/j.cmet.2011.04.011 21723506 PMC3130927

[B85] Wallace L. M. K. Theou O. Godin J. Andrew M. K. Bennett D. A. Rockwood K. (2019). Investigation of frailty as a moderator of the relationship between neuropathology and dementia in Alzheimer’s disease: a cross-sectional analysis of data from the Rush Memory and Aging Project. Lancet Neurol. 18, 177–184. 10.1016/S1474-4422(18)30371-5 30663607 PMC11062500

[B86] Waltz T. B. Fivenson E. M. Morevati M. Li C. Becker K. G. Bohr V. A. (2018). Sarcopenia, Aging and Prospective Interventional Strategies. Curr. Med. Chem. 25, 5588–5596. 10.2174/0929867324666170801095850 28762310 PMC5792375

[B87] Wang X. Su B. Lee H. G. Li X. Perry G. Smith M. A. (2009). Impaired balance of mitochondrial fission and fusion in Alzheimer’s disease. J. Neurosci. 29, 9090–9103. 10.1523/JNEUROSCI.1357-09.2009 19605646 PMC2735241

[B88] Watson K. Baar K. (2014). MTOR and the health benefits of exercise. Semin. Cell Dev. Biol. 36, 130–139. 10.1016/j.semcdb.2014.08.013 25218794

[B89] Webster S. J. Bachstetter A. D. Nelson P. T. Schmitt F. A. Van Eldik L. J. (2014). Using mice to model Alzheimer’s dementia: An overview of the clinical disease and the preclinical behavioral changes in 10 mouse models. Front. Genet. 5, 88. 10.3389/fgene.2014.00088 24795750 PMC4005958

