# Supplementary material for: Targeting Multiple Mitochondrial Processes by a Metabolic Modulator Prevents Sarcopenia and Cognitive Decline in SAMP8 Mice
Source: Front Pharmacol. 2020 Jul 31;11:1171. doi: 10.3389/fphar.2020.01171 (PMC7411305; doi:10.3389/fphar.2020.01171)
Supplement: Supplementary file 1 [file DataSheet_1.docx]

Supplementary Material

# Table S1

| **PD-0E7 mixture composition** | |
| --- | --- |
| L-Leucine | 23.46 |
| L-Lysine HCl (chlorhydrate) | 17.59 |
| L-Isoleucine | 11.73 |
| L-Valine | 11.73 |
| L-Threonine | 13.68 |
| L-Cysteine | 2.93 |
| L-Histidine | 2.93 |
| L-Phenylalanine | 1.95 |
| L-Methionine | 0.98 |
| L-Tyrosine | 0.00 |
| L-Tryptophan | 0.98 |
| Vitamin B1 (thiamine chlorhydrate) | 0.0137 |
| Vitamin B6 (piridoxine chlorhydrate) | 0.0166 |
| Citric acid anhydrous | 7.99 |
| Malic acid | 2.00 |
| Succinic acid | 2.00 |
| **Leucine:Isoleucine:Valine Ratio** | **2:1:1** |

Composition is expressed as percentage of total amount (%, g/100 g)

# Table S2

|  | **untreated** | **PD-0E7** |
| --- | --- | --- |
| Food intake (g) | 5.49 ± 0.43 | 5.52 ± 0.62 |
| Water intake (ml) | 6.58 ± 0.91 | 7.37 ± 1.11 |

Data are from measurements in 12-month-old mice (6/group). Values represent mean ± D.S.
